# Cellulose Nano Crystals (CNC) as Additive for a Bio-Based Waterborne Acrylic Wood Coating: Decay, Artificial Weathering, Physical and Chemical Tests

**DOI:** 10.3390/nano13030442

**Published:** 2023-01-21

**Authors:** Swati Tamantini, Sara Bergamasco, Florian Zikeli, Miha Humar, Massimo Cavalera, Manuela Romagnoli

**Affiliations:** 1 Department for Innovation in Biological, Agro-Food and Forest Systems, University of Tuscia, 01100 Viterbo, Italy; 2 Department of Wood Science and Technology, Biotechnical Faculty, University of Ljubljana, 1000 Ljubljana, Slovenia; 3 Finedin s.r.l., 73057 Taviano, Italy

**Keywords:** beech, spruce, wood durability, *Gloeophyllum trabeum*, *Trametes versicolor*, color, gloss, blue stain, FTIR, contact angle

## Abstract

Wood coatings prolong the service life of wood-based products, but they are usually of synthetic origin. The aim of the present article is to reduce the fossil-based compounds in a commercial waterborne acrylic coating by CNC addition and to test its performance. The coatings were applied on European beech and Norway spruce wood in order to test durability against *Gloeophyllum trabeum* (brown wood rot) and *Trametes versicolor* (white wood rot). Artificial weathering and blue stain, contact angle, physical tests (adhesion, impact and scratch test), chemical (FTIR) and morpho-anatomical analysis (SEM) were carried out. CNC addition increased viscosity, limiting the spreading of the coating into wood pores as visible after SEM observation, which reduced coating adhesion on the substrate. CNC improved fungal resistance as seen by a reduced mass loss and FTIR spectroscopy thanks to crosslinks formation, which reduced water sorption as well. Color change was not significant, and, on the other hand, glossiness was reduced but resulted as more homogeneous than control. CNC addition gave good results also in blue stain protection. CNC improved scratch resistance, but no visible change to impact was registered. CNC has promising results in coatings depending on wood and fungal species and presence of further commercial additives (biocides).

## 1. Introduction

Wood is one of the most important building materials, and it has been utilized for construction applications for several millennia [1]. Recently, the importance of wood as a building material has increased due to the development of new wood composites such as cross-laminated timber (CLT) [2,3,4] and increased environmental awareness in Europe and other continents [5]. This trend is likely to continue.

Wood in outdoor applications is exposed to the effects of various abiotic and biotic factors. Wood decay is vital in the natural processes of carbon circulation, but when the wood is used for commercial purposes, we need to slow this process down as much as possible [6]. Abiotic degradation factors in synergy with blue stain fungi predominately affect the aesthetic of wood. UV light causes the degradation of lignin to quinones that leach from wood during rainfall events. This results in a silver-grey color of the wood. Furthermore, in the presence of blue stain fungi, the resulting wood color is considerably darker [7]. On the other hand, white and brown rot fungi also affect the structural integrity of the wood through the release of enzymes, depolymerization of key wood ingredients and consumption of degradation products [8]. Fungal decay can result in a relatively fast and prominent loss of mechanical properties; thus, it needs to be controlled and stalled as much as possible [9].

Unfortunately, most of the European wood species do not have durable wood [10]; thus, we have to protect it to ensure the desired service life. In the middle of the 20th century, most solutions focused on biocidal treatments. The biocidal treatments aimed to make wood toxic to wood-degrading fungi and insects [11]. Due to health and environmental concerns and consumers’ increased environmental awareness, most biocides were removed from the market after introduction of the biocidal products directive [12] and, later on, of the biocidal products regulation by the European Parliament [13]. Therefore, recent developments focus on the non-biocidal methods of wood protection, such as the use of natural products [14,15,16,17] and wood modification [18].

Increasing the durability of wood by sustainable methods is still one of the major challenges. To this aim, the successful use of physical methods such as thermal modification [19,20] and other chemical treatments [21] has been recently reported, but the novel and greener generation of wood coatings offers new interesting opportunities. Coating technology provides surface protection, decorative finishes and commodities with special functions. To ensure protection from wood-inhabiting fungi, coatings are often composed of synthetic compounds with biocidal functions [22] that could be harmful to humans or the environment.

Therefore, more sustainable compounds were tested in the coating industry. An example is cellulose, which has always been used for its availability, renewability, biodegradability and low-cost production. This polymer has been intensively employed in clothing, cordages, pottery, porcelain, clay bricks and paper [23,24,25] as it tends to create an entanglement between its fibers, strengthening tissue structure. With the industrial evolution, cellulose applications reached biodegradable packaging, dietary fibers as supplements, supercapacitors and advanced to chemicals such as fuel or additives for transparent wood [24,25,26,27,28,29]. In addition, there has been a transition in cellulose particles from macro, micro to nano scale, because research discovered an enhancement in different properties such as high surface-to-volume ratio, high tensile strength and stiffness and surface tailor-ability via hydroxyl groups [30]. This trend can be seen by the fact that the cellulose market was estimated to expand and reach USD 41.5 billion by 2025 [31]. Further, cellulose nanocrystals (CNC) and nanofibrils (CNF) reached TRL9 and now are sold on the common market. CNC, as a coating additive, provides antimicrobial activity [32,33], especially if combined with essential oil [34]. On the other hand, studies on CNC antifungal activity are scarce, and none of them addresses coating applications [35]. Indeed, CNC showed an interesting improvement in physical properties, increasing mechanical resistance when used in coatings or adhesives formulations [36,37].

In this frame, the present paper aims to test if using CNC as additive in a waterborne acrylic coating could affect fungal growth. The chosen fungal species are two of the most common: a brown rot fungus (*Gloeophyllum trabeum* (Pers.) Murrill) and a white rot fungus (*Trametes versicolor* (L.: Fr.) Pilàt). Moreover, a characterization of the resulting coating is presented, namely coating viscosity, coating uptake and physical tests such as adhesion, impact and scratch test. The coatings were also tested under artificial weathering utilizing xenon exposure; therefore, color and gloss analyses were carried out, together with resistance to blue stain. The tests were conducted on European beech and Norway spruce wood, key wood species in central Europe. As those wood species are not durable, there is a great commercial interest in improving their durability to meet the requirements of the use-class three application (above ground, EN 335:2013 [38]), which was found to be one of the most important in the construction industry.

## 2. Materials and Methods

### 2.1. Materials

The commercial water-borne acrylic coating is based on poly (methyl acrylate/methyl methacrylate/butyl acrylate) copolymer dispersion, and it was provided by the company Finedin S.r.l. (Taviano, Italy). The solid content of the commercial coating is 34%, viscosity is 40–50” F/4, gloss at 60° is 75–85, while the pH is 8.5. The acrylic coating is transparent and provides mechanical protection of wood against impacts and scratches in indoor and outdoor conditions. The tests were carried out on the acrylic coating in two different situations: a pure acrylic formulation and one with industrial biocides, common on the market for preventing molds. The commercial biocides are: 5-chloro-2-methyl-2H-isothiazol-3-one (MCI) and 2-metil-2H-isotiazol-3-one (MIT) with ratio 3:1. Cellulose nanocrystals (CNC) = chemically: cellulose hydrogen sulfate sodium salt, were purchased from CelluForce Inc. (Montreal, QC, Canada). The spray-dried CNC powder has a bulk density of 0.7 g/m^3^, a moisture content of 4–6% and a particle size spanning from 1 to 50 μm. CNC crystallites (particle diameter: 2.3–4.5 nm, particle length: 44–108 nm, crystallite fraction: 0.88, crystallite density: 1.5 g/cm^3^) have a hydrodynamic diameter of 70 nm [37]. Particle diameter and length were determined via AFM, and crystallite fraction was determined using an XRD analysis; these three parameters were measured by the National Research Council of Canada. All the reported information about CNC was supplied by CelluForce Inc. (Montreal, QC, Canada).

The coatings were applied to different species: European beech wood (*Fagus sylvatica* L.) and Norway spruce wood (*Picea abies* (L.) H. Karst.), selected because they are common in the wood market and because of their low durability. In order to study the coatings, no anti-fungal effect should come from the wood itself. Wood samples were taken from one board, cut in the tangential direction and conditioned in the lab at 65% relative humidity (RH) and 20 °C for one year. The samples were prepared with different sizes due to the different analysis methods. Samples for the durability test have dimensions of 30 × 15 × 9 mm^3^ (L × T × R), as an adaptation of the standard EN 113:2021 [39,40]. Samples for blue stain, xenon exposure and contact angle have dimensions of 25 × 25 × 5 mm^3^, as the surface is essential in this kind of test. Samples for physical tests (impact, scratch and adhesion tests) have dimensions of 150 × 70 × 10 mm^3^. Sample dimensions were smaller than the ones required by the standards because of the low quantity of formulations produced, since the procedure is still at laboratory scale. The nutrient medium used for the decay test was potato dextrose agar (PDA) (Difco™, Franklin Lakes, NJ, USA).

### 2.2. Methods

#### Coatings Preparation and Application

Prior to adding the CNC to the commercial coatings, a gel of CNC and water at 7% (*w*/*w*) was prepared. The water was stirred (300 min^−1^) and heated at 60 °C, and, in the meantime, CNCs were gradually added to the water. This process lasted 60 min. In order to thoroughly mix the compounds, the stirring speed was raised to 750 min^−1^ and the heating to 100 °C for 30 min. The CNC gel was added to the liquid coatings to have a final CNC concentration of 2%.

The abbreviations used in the text are listed in Table 1.

Wood samples were dipped in the coating once, left for 5 s and dried for 24 h.

### 2.3. Coating Viscosity

After 2 days, the viscosity was measured following the standard EN ISO 2431:2019 [41]. A flow cup of 100 mL (F/4) capacity was used. Three measurements per formulation were recorded (12 total measurements).

### 2.4. Coating Uptake

The mass of the samples was measured using an electronic analytical scale (EP 320A, LOTRIČ Metrology Group, Selca, Slovenia). Samples were weighed before coating application and after the drying period of 1 day. Six samples with the dimension of 30 × 15 × 9 mm^3^ (96 total samples) and six samples with the dimensions 25 × 25 × 5 mm^3^ (96 total samples) per each treatment were tested (192 total samples).

### 2.5. Decay Test

The decay test (D) was performed according to a modified protocol of the standard EN 113:2021 [39].

Before starting, the mass of the coated samples was recorded, and then they were placed in the oven for 48 h at 60 ± 2 °C in order not to damage the coatings. The weight of the specimens was eventually recorded after the samples were removed from the oven.

PDA was prepared and poured into sterilized Petri dishes (Ø = 85 mm, h = 15 mm). The samples and the filled Petri dishes were then sterilized in an autoclave at a temperature of 121 °C and pressure of 150 kPa for 20 min. From this point on, everything was performed in a sterile environment using sterile equipment. Plastic meshes (HDPE) were placed over the PDA in the Petri dishes in order to prevent direct contact between the samples and the growth medium. Then, the Petri dishes were inoculated with a brown rot fungus (*Gloeophyllum trabeum* (Pers.) Murrill) and a white rot fungus (*Trametes versicolor* (L.: Fr.) Pilàt). The fungal collection of the Biotechnical Faculty, University of Ljubljana, Slovenia, provided the fungal cultures [42]. In total, 120 samples with the dimensions of 30 × 15 × 9 mm^3^ (6 samples per treatment) in 48 Petri dishes (3 samples per Petri dish) were exposed in an incubation chamber (T = 25 °C; RH = 85%) for 12 weeks.

After incubation, mycelium was carefully removed from the surface of the samples. Then, the samples were weighed before and after oven drying at 103 ± 2 °C for 48 h. Mass loss (Δ*m*) was calculated according to the following Equation (1) by [43]:

(1) $$\Delta m\;\left[\%\right]=\frac{m_{0}\;\left[g\right]-m_{1}\;\left[g\right]}{m_{0}\;\left[g\right]}100$$

where *m*_0_ is the oven-dry mass of the sample before fungal exposure, and *m*_1_ is the oven-dry mass of the sample after the inoculation and incubation of 12 weeks (units of measurement are in square brackets).

### 2.6. Color and Gloss Analyses, Artificial Weathering and Blue Stain Fungi

The artificial weathering (AW) process of the samples (25 × 25 × 5 mm^3^) was carried out using the Atlas SUNTEST XXL+chamber (Atlas Material Testing Technology, Mount Prospect, IL, USA), with the daylight glass filter on. Conditions of the chamber were an irradiance of 0.35 W/m^2^, an irradiance wavelength of 340 nm, the temperature of the chamber was 38 °C, and the RH was 55%, as stated in the standard ISO 16474-2:2013 [44]. The samples were exposed for 240 h. Color and gloss values were recorded before and after the coating application after 120 h and 240 h of xenon exposure. Six specimens per treatment were analyzed (48 total samples).

The color difference was calculated using the following Equation (2) [45]:

(2) $$\Delta E^{*}=\sqrt{{\left(L_{2}^{*}-L_{1}^{*}\right)}^{2}+{\left(a_{2}^{*}-a_{1}^{*}\right)}^{2}+{\left(b_{2}^{*}-b_{1}^{*}\right)}^{2}}$$

where: L* is the lightness (0 to 100); a* is the coordinate for red/green values (−120 to 120); b* is the coordinate for blue/yellow values (−120 to 120); Index 1 stands for the reference sample; Index 2 stands for the sample to analyze.

EN 152:2021 [46] standard was followed for the blue stain procedure. The specimens exposed to xenon lamps and those not exposed (25 × 25 × 5 mm^3^) were tested for the blue stain fungi (96 total samples). The test organisms were *Aureobasidium pullulans* (de Bary) G. Arnaud, and *Sclerophoma pithyophila* (Corda) Höhn. Samples were exposed to the blue stain fungi for six weeks as prescribed by the respective standard.

### 2.7. Optical Contact Angle

Optical contact angle (CA) measurements on the acrylic formulations were performed at room temperature using the optical tensiometer Attention Theta Flow (Biolin Scientific Group, Gothenburg, Sweden). Six samples per treatment were tested. A drop of 4 μL of distilled water was released on the coated samples, and the observation time was 60 s per specimen.

Data elaborations were conducted using the software OneAttension v4.1.2 (r9576), provided by Biolin Scientific Group (Gothenburg, Sweden).

### 2.8. Impact Test

The resistance to impact was determined following the standard ISO 4211-4:1988 [47]. A steel cylinder of 500 ± 5 g was placed at different heights (25 and 100 cm) for free fall onto a steel ball (Ø = 14 mm) placed on the surface of the coating. After impact, the surface was examined with a magnifier (10×) and the impact resistance of the coating was evaluated using numerical grades according to the standard. Three replicates were performed for each species and drop height.

### 2.9. Scratch Test

The scratch test was carried out following the standard EN ISO 1518-1:2019 [48]. A spring test pencil (Model 318, Erichsen GmbH & Co. KG, Hemer, Germany) was used for the purpose. A needle is inserted in it with a tip of a half-shaped sphere of 1 mm in diameter. The indent measurements were recorded at a load of 5 and 10 N. The scratch length was at least 60 mm, and the scratch speed was 30–40 mm/s. The scratch was performed perpendicular to the grain direction. The broadest indent width was recorded per each species and load.

### 2.10. Adhesion Test

Pull-off adhesion test was carried out according to the standard EN ISO 4624-2016 [49]. Aluminum dollies (Ø = 20 mm) were glued on the surface of the coatings using a 2-component polyurethane adhesive. After a curing period of 24 h, the coating around the dollies was carefully cleaned down to the substrate in order to isolate the glued zone from the rest of the coating layer. The tensile stress applied to peel off the wood surface coating was measured using a Defelsko Positest^®^ Adhesion tester (Defelsko instruments corporation, Ogdensburg, NY, USA).

### 2.11. FTIR Spectroscopy

One representative wood sample per coating formulation was analyzed using a Jasco IRT-7000 Irtron Infrared microscope (Jasco Corp., Easton, MD, USA) in order to investigate the surface of the coated samples using FTIR imaging. The number of scans was 300 per measurement point (5 × 5) and the aperture size was 10 × 10 μm^2^. Each specimen was analyzed twice: (I) a damaged area with hyphae presence and (II) a hyphae-free area, where the coating seemed clean and sound.

### 2.12. SEM

For scanning electron microscopy (SEM), samples were attached to aluminum stubs using carbon tape and sputter-coated with gold in a Balzers MED 010 unit. The observations were made by a JEOL JSM 6010LA electron microscope using secondary electrons (SE). The analysis was carried out on one representative decay test sample per coating to investigate the damage level.

### 2.13. Statistical Analyses

Data storing and elaboration were performed using MS Excel (Microsoft 365, Microsoft, Redmond, WA, USA). The statistical analyses were carried out using Minitab (v18.1, Minitab Inc., State college, PS, USA). Test of normality (Anderson–Darling method) was conducted in order to identify which hypothesis test fitted the data. In case of normality distribution, the ANOVA test was chosen, and a Tukey test was performed for the statistical significance. The influence of the CNC addition on the pure commercial coatings was assessed by principal component analyses (PCA) on coating uptake, color and gloss analyses. The most relevant results are shown.

### 2.14. Experimental Design

**Figure 1:**
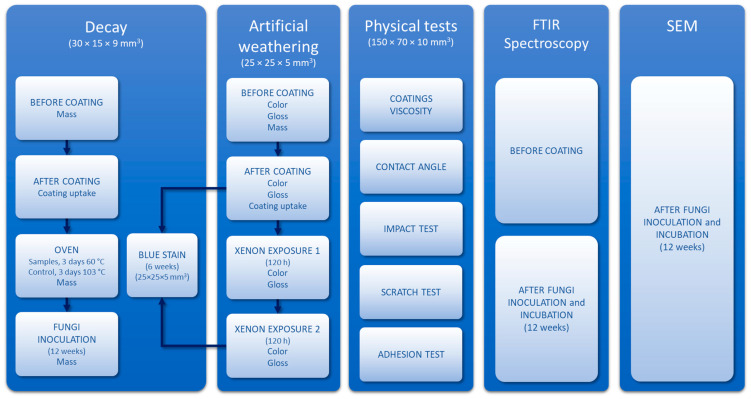
Experimental design of the present study.

The experimental design is summarized in Figure 1.

## 3. Results and Discussions

### 3.1. Coating Viscosity

Table 2 shows the results of the formulation’s viscosity measured after two days, performed at three samples for each test formulation. In this experiment, the viscosity parameter was used to define the difference between the reference acrylic coating with and without commercial biocides and the modified coatings by CNC addition.

As it is possible to see in Table 2, CA viscosity is a little higher than CAB. This difference is likely associated with the intended use. Coatings with biocides are usually applied as primers that need to penetrate deeper into the wood; thus, they are less viscous than other layers applied to the wood. However, CA+CNC viscosity is lower than CAB+CNC; in any case, CA+CNC and CAB+CNC viscosity is higher than CA (almost six times higher) and CAB (almost 20 times higher), respectively, and the difference is highly significant. Thus, the effect of CNC is more evident in CAB coating.

Thus, CNC addition caused a viscosity increase in the modified coating, in agreement with the literature [36,50]. Adding polymers with high molecular weight, such as cellulose, increases the viscosity of a liquid coating [51,52]. The addition of cellulose to paints and stains is also frequently used in several commercial products [53]. The increase in viscosity could cause problems with the sprayability of a coating and the diffusion of the material on the surface [50,54], but for waterborne acrylic coating, this issue can easily be resolved by the addition of water in order to adjust the viscosity [51,55].

### 3.2. Coating Uptake

In Table 3, it is possible to see that spruce samples had higher values than beech wood. A possible explanation is the higher surface roughness of the spruce samples, which absorb more liquid due to capillary tension [56] compared to smoother surfaces. Except for the durability (D) of beech samples coated with CAB+CNC and spruce samples used for AW when coated with CA+CNC, in all other cases, the samples coated with the modified coating by CNC show a higher coating uptake. The highest effect is in artificial weathering samples passing from CAB to CAB+CNC coating (about and more than double). The higher uptake in general in spruce is consistent with the viscosity results. Higher viscosity is always related to higher weight after dip coating application [57].

It is worth mentioning that beech wood samples for durability test coated with CAB and CAB+CNC show a high standard deviation in coating uptake.

### 3.3. Decay Test

**Figure 2:**
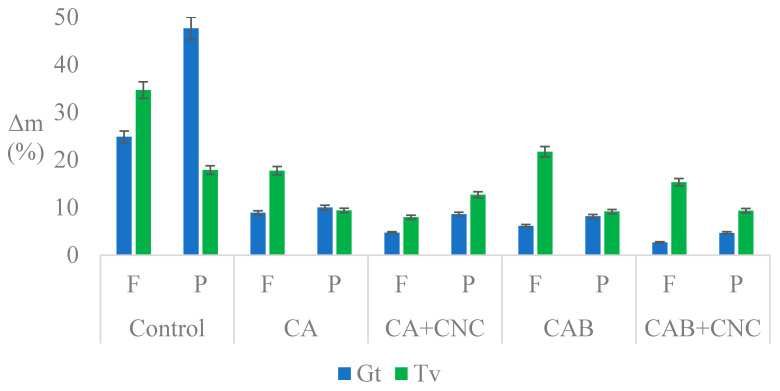
Mass loss (Δm) after the decay test of brown wood rot (Gt) and white wood rot (Tv). Control: Control sample (uncoated wood in oven at 60 °C for 3 days before fungi inoculation). F: *F. sylvatica*. P: *P. abies*. CA: Commercial acrylic waterborne coating. CA+CNC: CA with addition of 2% CNC gel. CAB: Commercial acrylic waterborne coating with biocides. CAB+CNC: CAB with addition of 2% CNC gel.

On average, the coating prevents fungal degradation. This can be seen from the mass losses, which are considerably lower for coated wood compared to non-coated references (Figure 2). Namely, the mass loss of coated wood was approximately three times lower (mean 9.83%, 6.18% for *G. trabeum*, Gt, and 11.36% for *T. versicolor*, Tv) than determined for the reference samples (mean 30.43%, 31.57% for Gt and 26.15% for Tv). High mass losses clearly indicate that the fungal strains were vital, and the wood was susceptible to fungal decay (see EN 350:2016 [58]). The negative effect of coating on fungal growth and decay can be ascribed to the fact that the coating acts as a membrane that limits hyphae penetration and to the possible presence of inhibitory compounds in the coating. The presence of CNC has a positive effect on the durability of coated wood against wood decay fungi.

Gt, as brown wood rot, had more effect on spruce (7.86%) samples than on beech (4.50%) ones. On the opposite, Tv, as white wood rot, was more effective on beech (12.56%) than spruce (10.15%). This finding agrees with the literature, which states that brown rot fungi are more frequent on softwoods, while white rot fungi are more effective degraders of hardwoods [59,60,61].

Regarding beech samples exposed to Gt, wood treated with CA and CNC blend exhibited higher mass loss than samples treated with CAB and CNC blend, respectively, meaning that biocides were effective on fungal growth. On the other hand, the situation for beech treated with Tv is the opposite, CAB and CAB+CNC had higher values than CA and CA+CNC, and so it appears the biocides did not affect Tv growth, even though in both cases (Gt and Tv), CNC seems to reduce the mass loss. CNC might cause additional crosslinking of the coating, which additionally limits penetration of the hyphae into wood.

When analyzing the results of the spruce wood exposed to Gt, wood treated with CA and CNC blend exhibited similar mass losses as samples treated with CAB, except for the spruce wood coated with CAB+CNC, which showed a very little value. Therefore, CNC and biocides had lower effect on Gt in spruce samples than beech ones, but there is a synergic effect of CNC and biocides in CAB+CNC. On the contrary, spruce wood exposed to Tv exhibited higher values in CA+CNC compared to CA. For spruce wood samples coated with CAB+CNC and inoculated with Tv, a mass loss comparable (approximately 9%) to the samples coated only with CAB was observed. In other words, CNC addition did not affect Tv growth in spruce wood.

Isothiazolones are biocides effective in rather low concentrations [62]. However, their efficacy against brown rot fungi has been considerably higher than against white rot fungi. As brown rot fungi are the key degrading organisms in construction applications [8], lower efficacy against white rot fungi was not considered problematic. The tolerance of white rot fungi to isothiazolones and other organic biocides has been predominantly associated with the fact that the structure of several organic biocides is similar to lignin, which makes them easily degradable [63,64].

The novel standard for the classification of wood in durability classes can be applied to a surface-modified coated and impregnated wood (EN 350:2016; CEN/TS 15083-1:2005 [58,65]). This classification can be based on the mass losses of wood after exposure to wood decay fungi. Non-treated beech and spruce wood can be classified as durability class (DC) 5 (not durable wood), the same as indicated in the respective standard. CA-treated beech can be classified as DC 4 (slightly durable), while CA treated spruce performs a bit better (DC 3—moderately durable), a durability class which is comparable to larch. Adding nanocellulose (CA+CNC) to the system results in higher durability classes, namely DC 2 for beech and DC 3 for spruce. The addition of biocide improves the classification of spruce (DC 2), while the durability classification of beech remains comparable to other systems.

### 3.4. Color and Gloss Analyses for Artificial Weathering and Blue Stain Fungi Evaluation

**Figure 3:**
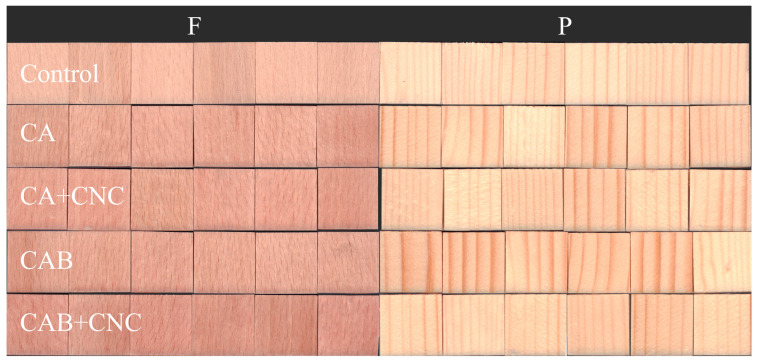
General appearance of the formulated coatings referred to uncoated wood. Control: Uncoated wood samples of beech (F) and spruce (P) wood. CA: Commercial acrylic waterborne coating. CA+CNC: CA with addition of 2% CNC gel. CAB: Commercial acrylic waterborne coating with biocides. CAB+CNC: CAB with addition of 2% CNC gel.

In Figure 3, the visual appearance of the treated wood samples before fungal exposure is illustrated.

**Figure 4:**
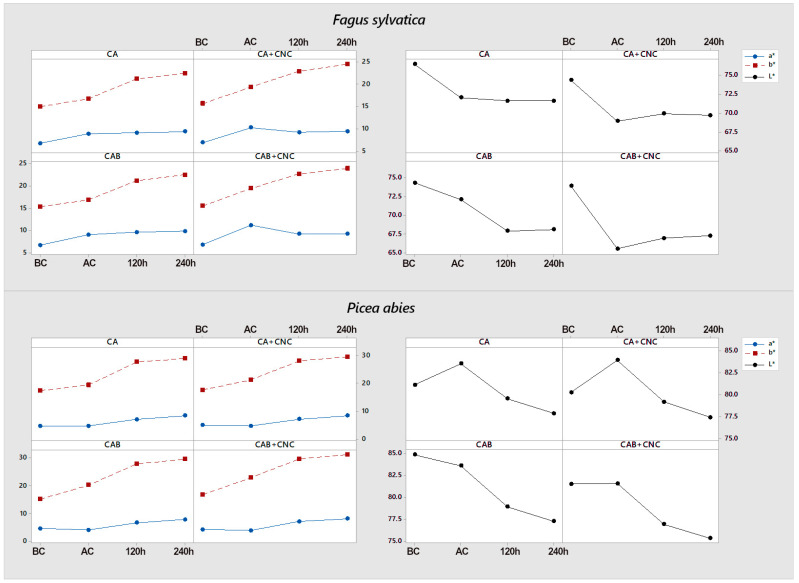
Color analysis results. a* (red-green) and b* (blue-yellow) coordinates on the left and L* (lightness) coordinate in the right for *F. sylvestris* and *P. abies*. BC: Before coating. AC: After coating. 120 h: After xenon exposure for 120 h. 240 h: After xenon exposure for 240 h. CA: Commercial acrylic waterborne coating. CA+CNC: CA with addition of 2% CNC gel. CAB: Commercial acrylic waterborne coating with biocides. CAB+CNC: CAB with addition of 2% CNC gel.

Beech wood is characterized by a higher value of a* coordinate compared to spruce, which indicates a more reddish color (Figure 4). Spruce wood is lighter than beech wood, and, after coating application (AC), it becomes lighter (increasing L* value). The values are higher in CA coating samples than untreated ones, whereas in CAB formulations L*, values decreased or were stable (Figure 4). On the beech samples, all acrylic formulation caused a decrease in L* values, especially the CNC modified coatings. Acrylic coating affects b* coordinate in both species. In fact, for all formulations, it increases from BC (before coating) to AC, turning the color to a yellower tone (Figure 4). Artificial ageing affects CIELAB coordinates. The variation is more evident in the first period from 0 to 120 h; as it was shown, the effect is usually much more remarkable after the first hours of exposure [19,66] (Table 4).

The color change geometry is reported in Table 4.

The coordinate b* increases from AC to 120 h (after xenon exposure for 120 h) and from 120 h to 240 h (after xenon exposure for 240 h), which means the wood acquires a yellowish tone. Yellowish tones are signs of UV-induced lignin degradation [67]. Relative to the a* coordinate in beech and spruce, it is possible to conclude that, generally, the values increase, indicating that the color changes to reddish tones, but the differences are not that remarkable, especially in beech wood from 120 h to 240 h (Figure 4). In particular, in beech wood, the formulations with CNC usually have a light decrease in the a* coordinate, meaning greener tones of the specimen surfaces (Figure 4).

Control samples, without any coating, always have a higher color difference relative to all other formulations. This means the coating protected the wood to some extent. After coating application, the color variation of the CNC blends was always higher than the one of the pure coatings in both beech and spruce wood. As it is possible to see in Table 4, color change is most pronounced in the first 120 h, and then it slows down for the next 120 h (240 h), which confirms the observation carried out in Figure 4. After the AW (120 h and 240 h in comparison with AC), spruce samples had consistently the highest variation in all four coatings. This is due to the wood species’ natural color: spruce color is lighter than beech; therefore, it is more sensitive to discolorations. Beech is a little more pigmented compared to spruce, and it is widely known that pigmentation is a protective feature against sunlight degradation [68,69,70,71,72]. Speaking of the AW, CNC blends are different depending on the wood matrix and the presence of biocides. Studies on artificially weathered CNC-reinforced coatings are few, but in any case, they report a UV resistance due to light scattering caused by the nanoparticles [55,73]. After the first 120 h, beech samples coated with CA+CNC and spruce ones coated with CAB+CNC had lower differences than CA and CAB, respectively.

**Figure 5:**
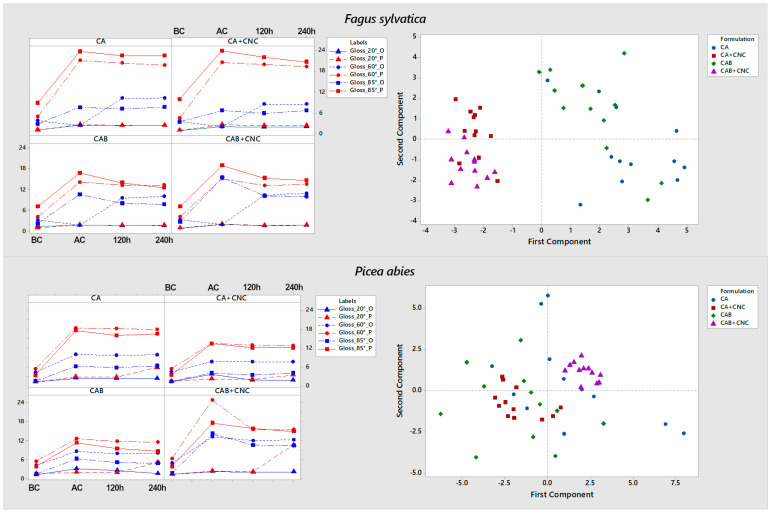
Gloss analysis. Mean plot (**left**) and PCA (**right**) for beech (F) and spruce (P) samples. BC: Before coating. AC: After coating. 120 h: After xenon exposure for 120 h. 240 h: After xenon exposure for 240 h. O: Gloss measurement orthogonal to wood grain. P: Gloss measurement parallel to wood grain. CA: Commercial acrylic waterborne coating. CA+CNC: CA with addition of 2% CNC gel. CAB: Commercial acrylic waterborne coating with biocides. CAB+CNC: CAB with addition of 2% CNC gel.

After coating application (AC), the gloss increased in all samples (Figure 5). Gloss parallel to wood grain is higher than the perpendicular one, as found in the literature [71]. The artificial weathering decreased the gloss as expected [71,74], notwithstanding that the values were equal to the AC values or even slightly higher in some cases. *P. abies* samples have higher gloss values than *F. sylvatica*.

Gloss parallel to grain is always lower in modified coating than reference, and the literature confirms this [50,57,75]. For the orthogonal direction, in the 20° angle, the gloss unit (GU) is lower in the coating with CNC than in the pure coatings, which is in accordance with the literature [50,57,75], but in the 60° and 85° angles, the values are higher than for the reference. Comparing AC samples to the ones after 120 h of xenon exposure (120h), the gloss decreased, except for the spruce samples coated with CA and the beech samples coated with CAB measured in the orthogonal direction, where the gloss is lower than before the xenon exposure. Gloss change from 120 h and after 240 h of xenon exposure (240 h) is not remarkable, and comparing AC samples to 240 h, the differences are negligible, sometimes in all formulations. PCA analyses highlighted two clusters in beech wood, this means gloss of CNC blend coated samples is statistically different from standard coatings. This is true also for spruce samples coated with CAB+CNC and, at some extent, also for CA+CNC in comparison with control samples. Therefore, CNC affected reduced gloss, in agreement with the literature [50]. The presence of biocides in coating produces a different behavior of CNC in affecting gloss measurement in spruce specimens because two different populations are observed in the opposite axes of PCA. On the other hand, CNC-based coatings with or without biocides in beech are more similar, and they are located in the same side of the PCA axes.

**Figure 6:**
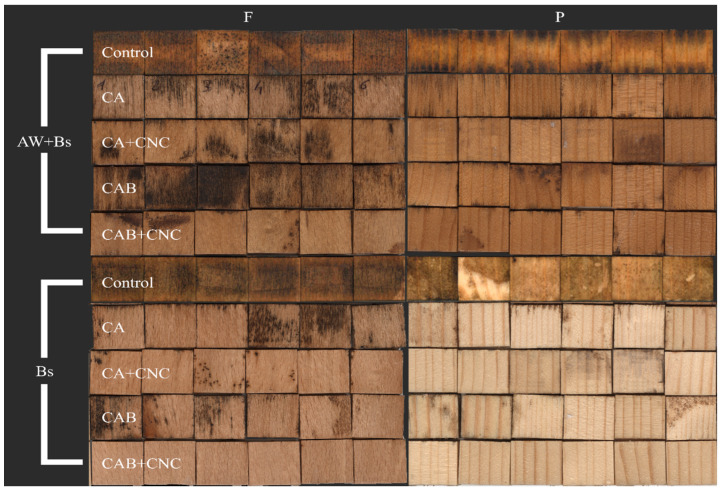
Blue stained samples. AW+Bs: Samples exposed to blue stain fungi after artificial weathering. Bs: Samples exposed to blue stain fungi only.

In Figure 6, the blue stain results are reported. As it is possible to see in Figure 6, the CNC addition helped the coating to tackle the fungi, especially in beech samples. Comparing the wood species, it seems spruce was more resistant than beech. 

Blue staining was performed on artificially weathered (AW+Bs) and non-weathered samples (Bs). Control specimens, regardless of weathering, were entirely covered with the blue stain fungi. The weathered samples were a bit more brownish, resulting from weathering and UV-induced degradation of lignin. All coatings have a positive effect on the performance of treated wood. The positive effect of coatings was more evident in spruce wood than in beech, because staining on spruce wood was limited to a few spots. Beech samples showed to be much more sensitive to blue staining attack. From Figure 6, a positive effect of nanocellulose was observed in weathered and non-weathered samples. Surprisingly, the nanocellulose was even more effective than the addition of biocides. Biocides were effective only in the presence of nanocellulose, where an even slightly synergistic effect was determined. The low efficacy of the respective biocides can be ascribed to the fact that isothiazolones are predominately effective against wood-degrading fungi and sapstain fungi [62]. Their efficacy against blue stain fungi has not been reported in the scientific literature [76]. The negative effect of weathering on the susceptibility of the wood against blue-stain fungi has been reported already. This phenomenon is ascribed to the leaching of the secondary metabolites from wood, degradation of the cellulose and hemicellulose into simple sugars. All these changes make wood more susceptible to fungal infestation. In addition to that, predominately UV radiation can result in the degradation of the biocides. UV degradation is more prominent at biocides applied superficially compared to the pressure treated wood [7].

### 3.5. Contact Angle

**Figure 7:**
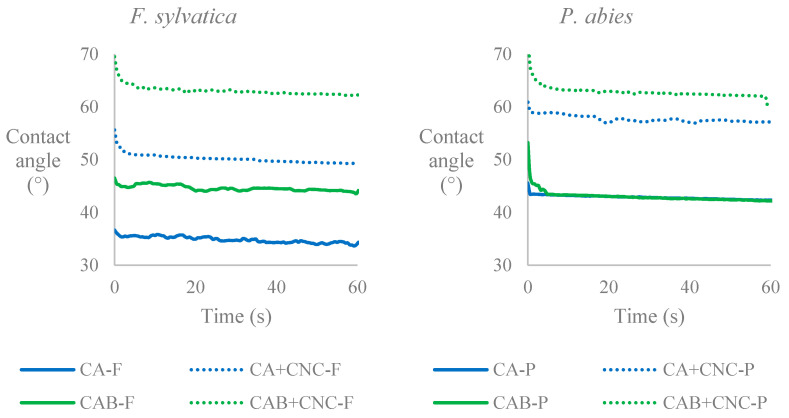
Contact angle analysis of the coatings applied on beech and spruce wood. CA: Commercial acrylic waterborne coating. CA+CNC: CA with addition of 2% CNC gel. CAB: Commercial acrylic waterborne coating with biocides. CAB+CNC: CAB with addition of 2% CNC gel.

Figure 7 reports the trend of the dynamic contact angle (60 s) for the studied coatings. In Figure 7, it is possible to see that CAB and CAB+CNC coated samples had the same values in both beech and spruce wood. Whereas CA and CA+CNC had lower values in beech samples than in spruce ones. This means CA coating and its CNC mixture rendered spruce samples more hydrophobic than beech samples. It could be the higher coating uptake, which made spruce wood more resistant to water impregnation. Regarding CNC, their addition increased the contact angle of both pure coatings and in both wooden species. CNC formulations display higher values than the ones reported in the literature [36,73,74], and this is probably due to the CNC gel addition to the pure coating instead of the direct addition of the CNC into the coatings; the gel was made in order to favor the CNC dispersion. CNCs are considered as mechanical resistance enhancers [73,77], and they are hydrophilic when applied by themselves [78]; therefore, the given hydrophobicity could be due to the established crosslinks between the matrix and the additive [79]. In fact, cellulose hydroxyl groups could have formed covalent bonds with acrylate groups of the coating [54,79,80,81], limiting the availability of the functional groups, which usually interact with water (hydroxyls).

### 3.6. Impact Test

Results of the impact test are reported in Table 5.

Apparently, there is no measurable variation due to the presence of CNC, even if, in the literature, CNC addition is reported as a mechanical resistance enhancer [50,54,57]. The issue is within the test methods: there are five levels of damage, where five means no damage and one means severely damaged. Therefore, sometimes the improvement is negligible or not visible. Since spruce is a softer species than beech wood [82], the sphere usually produces a more significant indent, as it is possible to see in the CA coating, but this fact does not happen in other cases.

### 3.7. Scratch Test

The results of the scratch test are reported in Table 6.

The scratch test gave good results and was in agreement with the literature [50,55,73]. In fact, except for the test with 10 N in the spruce samples, the CNC coating always had lower values, even if the differences were small.

### 3.8. Adhesion Test

The results of the adhesion test are reported in Table 7.

The modified formulations slightly decreased the adhesion of the coating on beech wood. In spruce, adhesion is lower too, but this time the difference is significant. The lower performances in adhesion could be due to the higher viscosity of both modified formulations, which could limit the spreading of the coating into the wood, as [55] also found out. The adhesion of a coating on the surface mainly depends on its diffusion and penetration into the wood structures [37,83,84].

Moreover, spruce values are half that of the beech ones; this means the substrate where the coating is applied plays an important role as well: spruce is a softer wood than beech; therefore, the adhesion resistance is lower.

### 3.9. FTIR Spectroscopy

**Figure 8:**
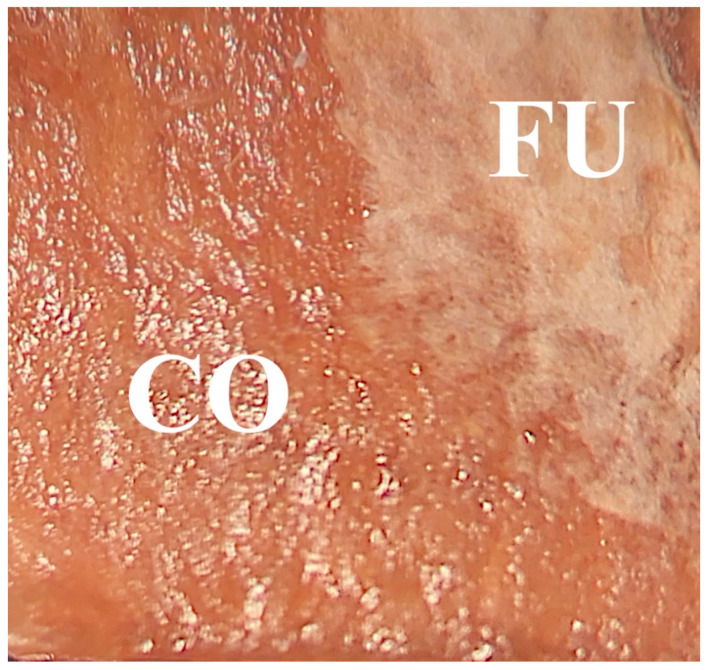
Spots for FTIR spectra acquisition. CO: Mycelium-free area. FU: Mycelium area.

Coated samples exposed to fungi (CA, CA+CNC, CAB, CAB+CNC) spots on the samples treated with one of the utilized fungi and showing no trace of fungal mycelium visible to the eye (CA-CO, CA+CNC-CO, CAB-CO, CAB+CNC-CO), as well as spots with visible fungal mycelium (CA-FU, CA+CNC-FU, CAB-FU, CAB+CNC-FU), were analyzed by FTIR microscopy (Figure 8).

**Figure 9:**
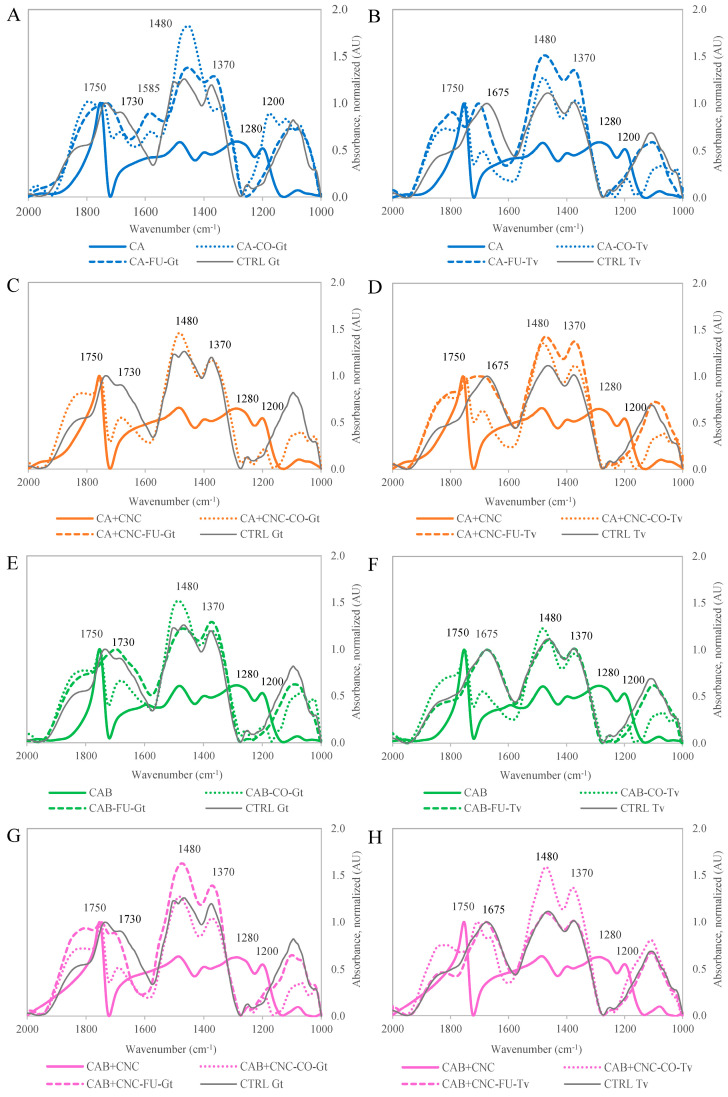
FTIR spectra of the coated wood samples. CTRL: Control samples exposed to wood rot. CA, CA+CNC, CAB, CAB+CNC: Coated wood samples not exposed to wood rot. CO: Spectra acquisition on coated wood samples exposed to wood rot, where the spot of measurement was mycelium free. FU: Spectra acquisition on coated wood samples exposed to wood rot on visible mycelium. (**A**) FTIR spectra of CA exposed to Gt. (**B**) FTIR spectra of CA exposed to Tv. (**C**) FTIR spectra of CA+CNC exposed to Gt. (**D**) FTIR spectra of CA+CNC exposed to Tv. (**E**) FTIR spectra of CAB exposed to Gt. (**F**) FTIR spectra of CAB exposed to Tv. (**G**) FTIR spectra of CAB+CNC exposed to Gt. (**H**) FTIR spectra of CAB+CNC exposed to Tv.

Figure 9 shows the FTIR spectra of the four used acrylic coatings formulations CA, CA+CNC, CAB and CAB+CNC, respectively. The acquired FTIR spectra of each FTIR microscopy measurement were averaged and eventually used for the overlays in Figure 9. Regarding the coated samples exposed to wood rot, besides the strongest IR band at 1750 cm^−1^, which is attributed to the C=O group of the acrylate repeating unit in the acrylate polymer, IR bands deriving from the underlying wood are visible at 1480, 1400, 1280 and 1200 cm^−1^, corresponding to cellulose, hemicellulose and lignin structural motifs, respectively. Similar observations were already reported in an earlier work of the group [36], but the literature about the topic is poor. Comparing the spectra of CA and CAB, the IR band around 1750 cm^−1^ becomes slimmer for the formulation with added biocide (CAB), and one different IR band at 1595 cm^−1^ is evident in the spectrum of CAB. Both of these differences could be attributed to C=O as well as C-N structural patterns of the used isothiazolinone type biocide. The addition of CNC to the respective formulations of CA and CAB (CA+CNC, CAB+CNC) leads to a broader IR band at 1750 cm^−1^ and, therefore, relatively elevated absorbance between 1680 and 1150 cm^−1^, which is attributable to the absorbance of the added CNC corresponding to characteristic IR bands of cellulose and nanocellulose structural motifs in this IR wavenumber range [33,85,86].

In Figure 9, the FTIR spectra of the coated wood samples, the coated wood samples after treatment with the fungi, as well as the respective spectra of the fungi grown on untreated wood are depicted for all four acrylic formulations and both fungi used.

For the first case of commercial acrylic coating (CA), both spectra CA-CO-Gt and CA-FU-Gt indicate the presence of grown fungus (Figure 9A). However, in the first case CA-CO-Gt, the spectrum profile is different from the one of *G. trabeum* grown on untreated wood (CTRL Gt) and exhibits higher absorbance at 1800, 1450 and 1200 cm^−1^, respectively. When analyzing the spot with visible mycelium of *G. trabeum*, the profile of the FTIR spectrum of CA-FU-Gt is becoming much more similar to the one of *G. trabeum* grown on untreated wood (CTRL Gt). This observation is quite surprising since no trace of mycelium was visible to the eye for the spectrum CA-CO-Gt, indicating that in this case, the wood structure is already colonized by the fungus. Both spectra CA-CO-Gt and CA-FU-Gt, respectively, exhibit a strong absorption band at 1585 cm^−1^, which could be attributable to a metabolite of *G. trabeum* exclusively in the presence of CA coating, since no IR absorption at this wavenumber was detected in the spectrum CTRL Gt.

In the case of CA treated with *T. versicolor*, a similar general situation was observed (Figure 9B). The spectrum of a spot without visible fungal mycelium (CA-CO-Tv) already shows the shape of the spectrum of *T. versicolor* grown on untreated wood (CTRL Tv), but still exhibits absorption at IR bands characteristic for the FTIR spectrum of the untreated coating at 1700 cm^−1^ and 1200 cm^−1^, respectively.

For the coating formulation CA+CNC, no spot with visible fungal growth on the sample surface could be identified. Thus, the respective FTIR spectrum of CA+CNC-FU-Gt is missing in Figure 9C.

However, the FTIR spectrum of CA+CNC-CO-Gt already shows a similar profile as it was observed for CA-CO-Gt, indicating the presence of fungal mycelium inside the wood structure even if it was not visible to the eye. The coating formulation CA+CNC treated with *T. versicolor* similarly showed fungal growth on the coated beech wood sample on both investigated spots with and without visible fungal mycelium (Figure 9D).

Even when applying the acrylic coating containing a biocide (CAB), the FTIR spectra show a profile that indicated some growth of *G. trabeum* (Figure 9E) as well as *T. versicolor* (Figure 9F) when analyzing spots where no fungal mycelium was evident. However, the IR band at 1750 cm^−1^ was still exactly at the wavenumber as for the untreated coating in contrast to the coating formulations CA and CA+CNC, respectively. This observation indicates that the coating formulation with added biocide CAB to some extent inhibited fungal growth on the coated wood. The spectrum of the colonized spots on the samples treated with CAB, however, showed a profile that was characteristic for the respective fungi, even if in the case of *G. trabeum* differences to the CTRL Gt fungi were detected, suggesting a certain stress caused on the fungi by the applied biocide. In the case of *T. versicolor* instead, the FTIR spectrum CAB-FU-Tv was almost identical to the CTRL Tv spectrum, confirming the more effectiveness of Tv in comparison with Gt on both wood species.

Analyzing the coating formulation CAB+CNC, the IR band at 1480 cm^−1^, which is characteristic for the acrylic coating formulation, remains dominant, and the IR band at 1750 cm^−1^ shows almost no shift, even when analyzing a sample spot with evident fungal growth of *G. trabeum* (Figure 9G). These two observations indicate that fungal growth on beech wood coated with CAB+CNC was significantly reduced for the case of *G. trabeum*. However, in the case of *T. versicolor*, the spectrum of CAB+CNC-FU-Tv was almost identical to the spectrum of CTRL Tv, while at the analyzed spot without evident presence of fungal mycelium, the spectrum was still dominated by the coating-specific IR band at 1480 cm^−1^. This indicates a certain but limited protective effect of CAB+CNC against attack from *T. versicolor* (Figure 9H). Conclusively, this could be explained by a synergistic effect of CAB coating and the added CNC, resulting in a certain inhibition of fungal growth of *G. trabeum* and *T. versicolor*, respectively.

CNC is usually added to coating for its mechanical property’s improvement. Creating a network with the matrix makes the resulting coating more flexible and, in the same moment, strong [78]. This crosslink between coating matrix, biocides and CNC limited the entrance of hyphae as well as water (see Section 3.5).

### 3.10. SEM

**Figure 10:**
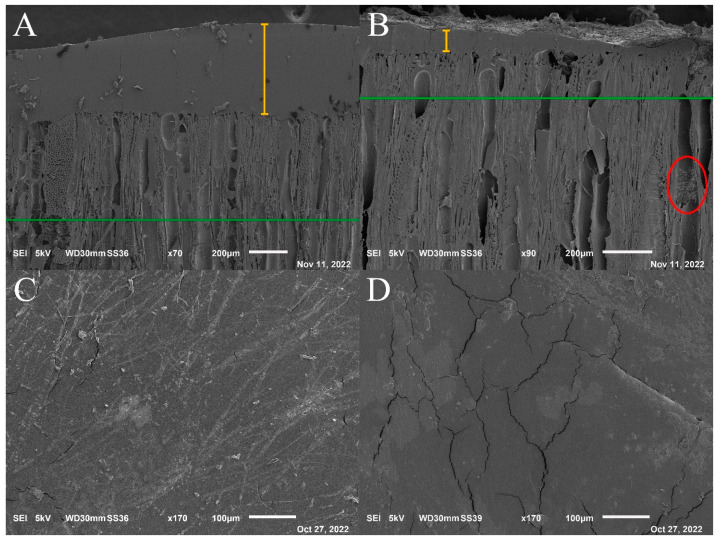
SEM observations. (**A**) CAB coated samples exposed to Tv. (**B**) CAB+CNC coated sample exposed to Tv. (**C**) CA+CNC coated samples exposed to Gt, stripes-shaped halo of the hyphae on the coating surface. (**D**) CA coated sample exposed to Gt, with light-grey halo on the coating surface, probably due to the metabolites produced by the fungus.

One representative sample per coating of beech wood decayed by Gt and Tv was chosen for the SEM analysis. Samples exposed to Gt exhibit the same behavior of the Tv ones, but with less damage, as stated in Section 3.3. From Figure 10, it is possible to see how homogeneous the hybrid coatings are.

Wood underneath thin or absent coating is completely damaged by the fungus (see red ellipse in Figure 10B indicating mycelium abundance), and, on the opposite, where the coating film was thick, the hyphae were poorly present (Figure 10A,B). Therefore, the irregular framing of wood samples by the coating gave the fungus the possibility to enter, confirming the findings of FTIR spectroscopy that the fungi affected coatings in some way and the wood as well.

On the coating surface, the same phenomenon can be seen: where the coating is thick (Figure 10A yellow line), hyphae are rare, where only wood is present or the coating is thin (Figure 10B, yellow line), the hyphae are big, branched and copious. Light grey trails on the coating surface are just the halo of the hyphae, which fell after the samples were oven dried (Figure 10C). Clusters of light grey areas on the wood surface could be the fungus metabolites, but this is not certain (Figure 10D). 

Figure 10A,B also show the coating penetration (green line), which spans from 200 to 500 μm. The SEM analysis confirmed what was stated in the coating viscosity and adhesion test paragraphs, namely, the higher viscosity of CNC blends reduced the diffusion of the coating into the wood interface. In fact, the penetration into the wood is lower for the modified coatings than for the reference coatings.

## 4. Conclusions

CNC addition caused modifications in the performance of the original coatings, but the result depends on the type of wooden substrate (in this case beech or spruce) and by the presence of synthetic biocides.

About durability, it can be assessed that commercial coatings already increased wood resistance to fungal decay acting like a shield against fungal mycelium. However, CNC blend resulted in a slight improvement, but the performance depends on the type of fungus because Tv produces more damage than Gt, generally. Adding nanocellulose (CA+CNC), the coating system results in higher durability classes, namely DC 2 for beech and DC 3 for spruce. The addition of synthetic biocides in the commercial coating (isothiazolones) improves the classification of spruce (DC 2) if CNC is present, while the durability classification of beech remains comparable to other systems. The good performance in the durability test is also supported by a reduced water uptake, which makes wood less sensitive to moisture increasing.

CNC addition slightly modifies color. In fact, there is no real change in a* and b* coordinates, but, in L*, the effect depends on the type of wood, on the presence of synthetic biocides in the acrylic coating, which interact with CNC, and on the aging effect. In beech, CNC makes wood darker than controls, but, after aging, CNC blends exhibit a behavior similar to the reference. In spruce, CA and CA+CNC trends are similar, but CAB+CNC is darker than CAB, due to a probable interaction of CNC with the added biocide to the synthetic formulation. Gloss becomes more homogeneous adding CNC in both spruce and beech, but, in spruce, the effect is different depending on the presence of synthetic biocides on the acrylic formulation.

Viscosity increases after CNC addition, and it could be a hurdle because it can limit coating penetration in wood pores, lowering film adhesion. In order to overcome this issue, the addition of solvent could reduce viscosity and improve coating penetration in wood, considering the industrial perspective. Impact test showed no real difference between commercial and formulated CNC coatings; on the other hand, scratch resistance is improved in CNC blends.

Furthermore, FTIR proved that fungal mycelium damaged coatings anyway to a certain extent, but the crosslink between CNC and the acrylic matrix limited the hyphae spreading throughout the wood. Thanks to SEM, FTIR spectroscopy results were confirmed, namely, that the fungus degraded the paint and passed into the wood in the thinnest parts of the coating.

In general, CNC showed promising results. It improved coating hydrophobicity, resistance to wood decay, blue stain and scratching, without sacrificing general appearance. This makes CNC an outstanding material to be appreciated in the coating industry, and, since it is at a commercial level already, CNC-based coatings could be a possibility in the near future.

## Figures and Tables

**Table 1:** Formulations and abbreviations of the coatings used in the text.

Abbreviation	Formulation
CA	Commercial acrylic coating
CA+CNC	CA with the addition of 2% gel of CNC
CAB	Commercial acrylic coating with biocides
CAB+CNC	CAB with the addition of 2% gel of CNC

**Table 2:** Viscosity values for the formulated coatings. CA: Commercial acrylic waterborne coating. CA+CNC: CA with addition of 2% CNC gel. CAB: Commercial acrylic waterborne coating with biocides. CAB+CNC: CAB with addition of 2% CNC gel. Statistical differences are reported as: *** *p* < 0.001.

	Viscosity
CA	1′43”
CA+CNC	12′22” ***
CAB	1′15”
CAB+CNC	20′ ***

**Table 3:** Coating uptake (%) for the decay test (D) and for the artificial weathering (AW) samples. CA: Commercial acrylic waterborne coating. CA+CNC: CA with addition of 2% CNC gel. CAB: Commercial acrylic waterborne coating with biocides. CAB+CNC: CAB with addition of 2% CNC gel. Mean values that do not share a letter (same column) are significantly different.

	Coating Uptake (%)			
Sample	D		AW	
Wood Species	*F. sylvatica*	*P. abies*	*F. sylvatica*	*P. abies*
CA	10.39 ± 0.83 A	15.27 ± 0.89 B	13.28 ± 0.82 F	36.33 ± 2.26 I
CA+CNC	13.52 ± 1.21 A	17.70 ± 1.76 C	20.32 ± 1.79 G	25.45 ± 2.05 L
CAB	12.27 ± 8.20 A	13.63 ± 1.05 D	12.22 ± 0.81 F	17.16 ± 0.90 M
CAB+CNC	10.83 ± 9.54 A	22.67 ± 2.62 E	27.34 ± 3.29 H	37.18 ± 3.90 I

**Table 4:** Color difference results. BC: Before coating. AC: After coating. 120 h: After xenon exposure for 120 h. 240 h: After xenon exposure for 240 h. Control: uncoated wood, as control for artificial weathering (AW).

	ΔE* (BC-AC)		ΔE* (AC-120 h)		ΔE* (120 h–240 h)	
	*F. sylvatica*	*P. abies*	*F. sylvatica*	*P. abies*	*F. sylvatica*	*P. abies*
Control			13.0 ± 0.9 ^a^	20.0 ± 1.7 ^a^	4.1 ± 0.5 ^b^	5.5 ± 0.4 ^b^
CA	5.3 ± 1.1	3.9 ± 1.1	9.2 ± 0.8	15.7 ± 1.4	2.1 ± 0.4	4.0 ± 0.6
CA+CNC	7.2 ± 1.3	5.5 ± 1.0	8.1 ± 0.8	16.2 ± 1.2	2.5 ± 0.3	4.2 ± 0.7
CAB	3.7 ± 1.1	5.7 ± 0.5	7.6 ± 1.5	15.6 ± 1.6	2.7 ± 0.6	4.5 ± 0.8
CAB+CNC	10.3 ± 1.6	6.6 ± 1.1	8.7 ± 0.7	13.4 ± 1.4	2.3 ± 0.3	3.9 ± 0.5

^a^ ΔE* is the difference between uncoated wood and after 120 h of exposure. ^b^ ΔE* is the difference between uncoated wood and after 240 h of exposure. CA: Commercial acrylic waterborne coating. CA+CNC: CA with addition of 2% CNC gel. CAB: Commercial acrylic waterborne coating with biocides. CAB+CNC: CAB with addition of 2% CNC gel.

**Table 5:** Level of damage (ISO, 4211-4:1988) for the impact test on the acrylic coatings. The sphere was dropped at two different heights (25 and 100 cm).

	Level of Damage			
Species	*F. sylvatica*		*P. abies*	
Height (cm)	25	100	25	100
CA	4	4	3	3
CA+CNC	4	4	4	4
CAB	4	4	4	4
CAB+CNC	4	4	4	4

CA: Commercial acrylic waterborne coating. CA+CNC: CA with addition of 2% CNC gel. CAB: Commercial acrylic waterborne coating with biocides. CAB+CNC: CAB with addition of 2% CNC gel.

**Table 6:** Scratch test (EN ISO, 1518-1:2019) results.

	Maximum Indent Width (mm)			
Species	*F. sylvatica*		*P. abies*	
Load (N)	5	10	5	10
CA	0.4	0.6	0.7	1.2
CA+CNC	0.3	0.4	0.6	1.2
CAB	0.4	0.5	0.5	1.2
CAB+CNC	0.3	0.4	0.4	1.2

CA: Commercial acrylic waterborne coating. CA+CNC: CA with addition of 2% CNC gel. CAB: Commercial acrylic waterborne coating with biocides. CAB+CNC: CAB with addition of 2% CNC gel.

**Table 7:** Results of the adhesion test (EN ISO, 4624:2016) for the two wood species.

	Adhesion Stress (MPa)	
Species	*F. sylvatica*	*P. abies*
CA	7.80 ± 0.46	4.77 ± 0.11
CA+CNC	7.31 ± 0.62	3.54 ± 0.50 *
CAB	7.74 ± 0.42	4.94 ± 0.37
CAB+CNC	7.17 ± 0.38	3.58 ± 0.30 *

CA: Commercial acrylic waterborne coating. CA+CNC: CA with addition of 2% CNC gel. CAB: Commercial acrylic waterborne coating with biocides. CAB+CNC: CAB with addition of 2% CNC gel. Statistical differences are reported as: * *p* < 0.05.

## Data Availability

The data presented in this study are available on request from the corresponding author.
